# Physics-Informed Slope Dynamics for Low-Computational Intelligent Diagnosis and Fault-State Evaluation in Spacecraft Propulsion

**DOI:** 10.3390/s26216784

**Published:** 2026-10-27

**Authors:** Chin-Sheng Chen, Shih-Yu Li, Chin-Chun Chang, Cheng-Yen Yeh, Wei-Chieh Li, Lap-Mou Tam

**Affiliations:** 1 Graduate Institute of Automation Technology, National Taipei University of Technology, Taipei 10608, Taiwan; saint@ntut.edu.tw; 2 Graduate Institute of Manufacturing Technology, National Taipei University of Technology, Taipei 10608, Taiwan; 3 Department of Computer Science and Engineering, National Taiwan Ocean University, Keelung 202301, Taiwan; 4 Department of Mechanical Engineering, National Taipei University of Technology, Taipei 10608, Taiwan; t111300229@ntut.org.tw; 5 Master Program, Graduate Institute of Mechatronic Engineering, National Taipei University of Technology, Taipei 10608, Taiwan; t113408089@ntut.org.tw; 6 Institute for the Development and Quality, Macao 999078, China; fstlmt@um.edu.mo; 7 Department of Electromechanical Engineering, Faculty of Science and Technology, University of Macau, Macao 999078, China

**Keywords:** physics-informed intelligent diagnosis, spacecraft propulsion system, pressure-slope dynamics, solenoid valve fault, low-computational complexity

## Abstract

**Highlights:**

**What are the main findings?**

- A physics-informed pressure-slope framework achieved 100% classification accuracy for normal, solenoid-valve-fault, bubble-fault, and unknown conditions.
- Recursive slope matching estimated faulty-valve opening ratios with an MAE of 0.59% and a maximum error of 0.92%, including cases from an unseen spacecraft.

**What are the implications of the main findings?**

- The proposed slope-based framework provides interpretable fault diagnosis and valve-state estimation with low computational demand, supporting real-time implementation.
- The method offers a lightweight approach for predictive maintenance in spacecraft propulsion and can potentially be extended to other fluid-control systems.

**Abstract:**

This study addresses the critical challenge of propulsion health assessment in space missions by proposing a novel physics-grounded framework for fault-state evaluation and valve-opening ratio estimation. Inspired by similarity analysis and physics-based feature design, the method leverages transient pressure–time slopes as a core physical feature, paired with valve-opening parameters, for efficient anomaly classification. Validation used the PHMAP 2023 Data Challenge dataset, replicating a 2 MPa water-propellant spacecraft propulsion system monitored by seven pressure sensors at 1 kHz over three valve open–close cycles. The framework extracts pressure-slope features from transient water-hammer behaviors, establishes a deterministic mapping to predict actual valve openings during solenoid valve faults, and distinguishes normal, solenoid-valve-fault, and bubble-contamination conditions. It achieved 100% classification accuracy on the test dataset, including unseen spacecraft and novel fault modes, with a maximum opening-estimation error of merely 0.92%. Combining strong interpretability with low computational complexity, the physics-driven approach circumvents the heavy resource demands of traditional models and offers high potential for real-time onboard implementation. It bridges physical principles and data-driven execution, providing critical technical support to enhance mission reliability and autonomous maintenance in satellite propulsion and unmanned aerospace vehicles.

## 1. Introduction

In recent years, the health monitoring of aerospace propulsion systems (PHM) has evolved from simple fault alarms toward an integrated framework emphasizing interpretability, predictability, and maintenance planning. Lee et al. (2014) [1] conducted a review and stated that the core tasks of PHM can be divided into three layers—anomaly detection, fault classification, and degradation assessment—spanning methodologies from signal processing and statistics to machine learning. Among these, converting domain knowledge into engineered features often ensures robust performance, even when data are limited or noisy. In terms of evaluation, Saxena et al. (2010) [2] proposed a set of offline prognostic performance metrics, which has since become a common language for comparing methods across subsequent studies.

Regarding data sources and task definitions, the Fourth Asia Pacific Conference of the PHM Society 2023 (PHMAP 2023) [3] Spacecraft Propulsion Data Challenge provided a concrete scenario: based on seven pressure sensors (P1–P7) and four solenoid valves (SV1–SV4), the dataset covers normal operation, bubble contamination, and solenoid-valve-opening faults, and requires maintaining both classification and regression performance even on an unseen spacecraft (Spacecraft #4). The challenge highlighted two critical issues: first, domain shift causing distributional discrepancies and, second, the need to jointly handle classification and opening-ratio estimation under constraints of real-time operation and interpretability. The PHMAP 2023 study by Minami and Lee [4] demonstrated the effectiveness of a similarity-based model combined with physics-inspired features: system states were first classified via similarity measures, and then valve openings were estimated using pressure-drop features, achieving both generalization and computational efficiency.

Methodologically, time-series similarity alignment techniques (e.g., Dynamic Time Warping, DTW) have long been used in PHM for matching and distance measurement, capable of handling phase shifts and rate mismatches. However, their computational cost can be high, making them less suitable for real-time or edge-device applications. To reduce complexity and enhance interpretability, recent trends have shifted toward physics-informed feature engineering—mapping fluid-dynamic mechanisms into computable indicators such as instantaneous pressure drops at valve opening, initial slopes, and recovery time constants. For propulsion systems, phenomena like water-hammer effects, damping oscillations, and recovery dynamics directly manifest in the local slope of the pressure–time curve. Thus, using “slope” as a first-derivative feature captures the earliest anomaly signatures at the millisecond scale and is particularly sensitive to bubble-induced sudden pressure dips or slower recovery due to insufficient valve opening. Brennen (1995) [5] provided the classic foundation of cavitation and bubble dynamics, explaining how bubble formation, growth, and collapse alter the local pressure field, supporting slope and pressure-drop magnitude as effective indicators for detecting bubble contamination.

Meanwhile, deep learning has been increasingly applied to valve and hydraulic fault diagnosis [6,7,8,9,10,11,12,13], employing one-dimensional convolutions, temporal attention, and graph-structured models. Its strength lies in automatic feature learning and cross-condition fitting. However, when data are limited, real-time response is needed, or mechanistic interpretability is required, purely black-box models often suffer from overfitting and high deployment costs. Consequently, combining simple, physically meaningful low-dimensional features with data-driven comparison or regression when necessary offers a practical trade-off: the former ensures interpretability and fast inference, while the latter compensates for nonlinear mapping and domain adaptation. The PHMAP 2023 challenge’s measurement design (1 kHz sampling, 1.2 s duration, three cycles) also enables robust feature segmentation: the transient region between 0.1–0.4 s exhibits the most significant pressure oscillations, whereas the initial 0–0.1 s interval most clearly reflects changes in flow area and velocity, serving as a key window for slope and pressure-drop metrics.

Previous studies have discussed PHM strategies for aircraft control actuators and spacecraft-propulsion anomaly detection [14,15]. Regarding generalization, domain-shift effects can be mitigated through normalization, hierarchical similarity, or minimal target-domain calibration, while unknown-fault detection can rely on distance thresholds, uncertainty estimation, or open-set recognition. For evaluation, beyond classification accuracy, regression tasks must track metrics such as maximum MAE, segment-wise error distribution, and sensitivity at critical opening ratios (0%, 25%, 50%, 75%). In summary, existing studies converge on a consensus: physics-based low-dimensional indicators such as slope and pressure drop provide a robust and efficient diagnostic foundation for high-pressure, fast-transient systems like spacecraft propulsion. When combined with moderate similarity learning and polynomial regression or lightweight models, these features enable cross-domain recognition and precise valve-opening estimation while preserving interpretability. This perspective underpins the theoretical rationale and design principles of the slope-comparison and recursive-matching framework proposed in this study [16], explaining its robustness even under unseen spacecraft and unknown-fault conditions.

Recent research has addressed several complementary aspects of prognostics and health management (PHM) and intelligent fault diagnosis. Solís-Martín et al. [17] developed PHMD, an open-source tool that facilitates access to PHM datasets. Dai et al. [18] proposed a framework combining feature fusion and multilabel classification for the simultaneous diagnosis of control-valve faults, with consideration of fault similarity. Wang et al. [19] introduced a knowledge–data synergy approach to recognize previously unseen combinations of faults in sucker-rod pumping systems under a zero-shot setting. Huang et al. [20] presented a dynamic graph meta-learning approach that exploits spatial dependencies among multiple sensors for cross-category, small-sample fault diagnosis in railway turnout actuators. Li and Fetene [21] proposed a hybrid Graph Convolutional Network and Transformer Encoder model for lithium-ion battery temperature prediction, demonstrating improved accuracy over standalone models and its potential to enhance battery thermal management system reliability. Li and Gu [22] illustrated a smart fault-detection approach using chaotic mapping and feature extraction techniques to identify different ball-bearing fault conditions, improving real-time fault classification accuracy and efficiency. Li et al. [23] constructed a bearing fault-detection approach integrating fractional-order chaotic mapping and feature extraction techniques, achieving an average detection accuracy of 94.4% across different bearing fault conditions. These studies illustrate developments in dataset accessibility, simultaneous and composite fault diagnosis, and learning from limited fault samples. The present work addresses a different setting by using physically interpretable pressure-slope features for fault-state classification and valve-opening estimation in a simulated spacecraft propulsion system.

With the rapid advancement of modern aerospace technology, spacecraft propulsion systems have become indispensable to mission success, with their stability and reliability directly determining operational outcomes. Within these systems, solenoid valves regulate the release and flow path of propellants, making their performance critical to proper operation. Any anomaly—such as inaccurate opening ratios or flow blockage caused by bubble ingress—can lead to thrust loss, oscillations, or even mission interruption and failure. Existing approaches to solenoid-valve condition assessment generally fall into two categories: physics-based models, built upon fluid-dynamic and electromagnetic principles for interpretability, and data-driven models, which leverage machine learning to extract complex behaviors from large sensor datasets. The former offers clear physical meaning but struggles with nonlinearities and uncertainties, while the latter handles nonlinear relationships well but risks overfitting and lacks interpretability, posing challenges for safety-critical aerospace applications.

Inspired by the recent literature [16], which successfully combined similarity analysis with physics-based feature design to achieve notable results in propulsion health assessment, this study proposes a novel slope-comparison-based fault-state evaluation method. The approach analyzes the slope of pressure–time variation as a core physical feature, paired with valve-opening parameters, to identify abnormal states and estimate opening ratios, balancing interpretability with computational efficiency. The experimental validation uses the PHMAP 2023 [3] Data Challenge dataset, which simulates a water-propellant spacecraft propulsion system [5] operating at 2 MPa, controlled by four solenoid valves (SV1–SV4) and monitored by seven pressure sensors (P1–P7) at 1 kHz. The dataset encompasses multiple anomaly scenarios, including bubble contamination (at BV1 or BP1–BP7), solenoid-valve faults, and unknown anomalies, offering a rich basis for model development and verification.

This research aims to develop a physics-grounded analytical framework capable of efficient classification and accurate prediction by using pressure-slope dynamics for fault identification and opening-ratio estimation. The proposed method not only bridges the gap between traditional physical and data-driven models but also offers low computational demand, making it suitable for future applications in satellite propulsion and unmanned vehicles. Ultimately, it contributes to enhancing mission reliability and autonomous maintenance capabilities in aerospace and intelligent systems.

The main objectives of this study are as follows:

1. Slope Feature Extraction: Identify the first pressure drop within a specified time interval and compute the pressure-change slope by combining the corresponding time and initial pressure state. This slope serves as a physics-based feature for health state identification.
2. Opening Prediction via Slope: Using slope features obtained under various valve-opening conditions in the training set, establish a mapping between slope and opening ratio to estimate the actual valve opening during solenoid valve faults in the test cases.
3. Physics-Driven, Low-Computational Method: Propose a purely physics-based classification and prediction approach that requires minimal computational resources, effectively distinguishing between normal, solenoid-valve-fault, and bubble-fault conditions while estimating fault opening ratios and enhancing computational efficiency.

The dataset used in this study originates from the competition dataset released by the Prognostics and Health Management Asia-Pacific Conference 2023 (PHMAP 2023). This dataset was generated through numerical simulation to reproduce a complete spacecraft propulsion system (PHM), using water as the working fluid instead of actual propellants, which are typically volatile and highly pressurized. This substitution enables the safe and controlled simulation of real pressure dynamics during propulsion processes.

Before operation, the fluid is pressurized to 2 MPa (approximately 20 kg/cm^2^) to emulate the steady-state tank pressure conditions of a spacecraft propulsion system. Subsequently, four independently controlled solenoid valves (SV1–SV4) perform alternating open–close operations, replicating the thrust control cycles that occur during real propulsion phases.

To monitor pressure variations throughout different pipeline segments, seven pressure sensors (P1–P7) are installed along the main flow path. Each sensor is positioned at a specific location corresponding to different valves and branches, allowing real-time recording of pressure fluctuations induced by valve operations. In addition, the experimental setup includes several Bubble Points (BVs/BPs)—specifically at BV1 and BP1–BP7—where bubbles can be deliberately introduced. These points simulate abnormal conditions such as gas ingress or liquid vaporization in the pipelines, enabling analysis of how bubble contamination affects pressure response and alters system characteristics.

All sensors record synchronously at a sampling rate of 1 kHz, ensuring millisecond-level temporal resolution for capturing transient pressure behaviors. Each experiment spans 0–1200 milliseconds, resulting in approximately 1200 high-resolution samples per case, fully covering three valve open–close cycles. Such fine-grained data clearly reveal phenomena like rapid pressure drops, peaks, oscillations, and recovery processes, providing a solid foundation for slope computation, feature extraction, and anomaly detection. Overall, the dataset combines physical realism with statistical diversity, making it well-suited for developing and validating intelligent diagnostic models for spacecraft propulsion health monitoring.

**Figure 1:**
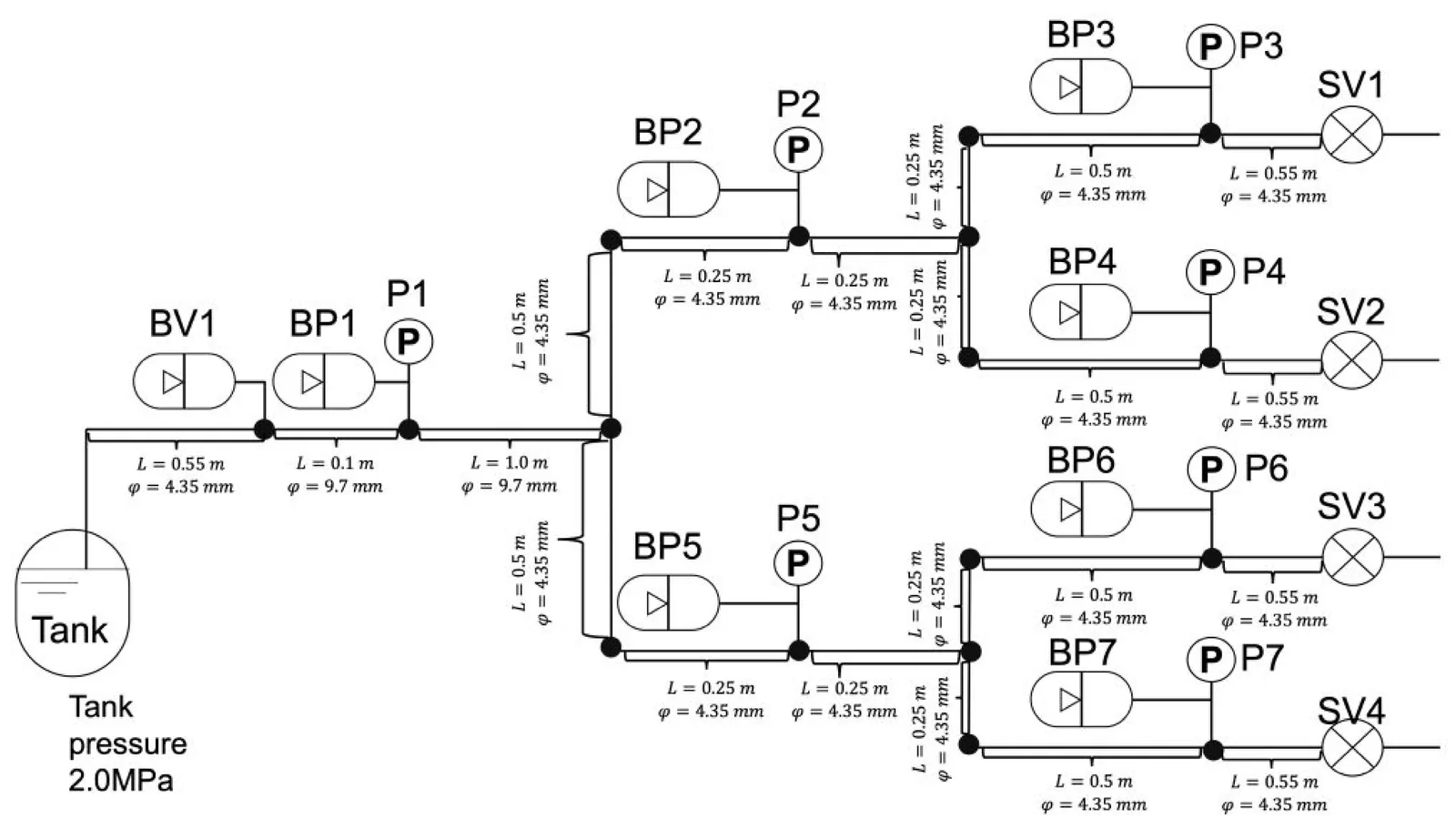
Schematic diagram of the experimental propulsion system [3].

Figure 1 illustrates the experimental propulsion system used in this study. The system was designed to safely and accurately emulate real spacecraft propulsion operations. Water was selected as a substitute fluid to replicate the motion characteristics of actual propellants. It is compressed to 2 MPa within a high-pressure storage tank to simulate the typical operating environment of space missions.

In terms of pipeline configuration, the system includes four controllable solenoid valves (SV1–SV4) to simulate the release and closure of liquid propellants in a thruster. The rapid switching of these valves generates pronounced water hammer effects, resulting in intense pressure fluctuations—precisely the transient behaviors that this study focuses on observing and analyzing.

To precisely capture the system’s dynamic response, seven pressure sensors (P1–P7) were installed at different positions along the pipeline to record real-time pressure variations. Each sensor operated at a sampling frequency of 1 kHz, and each experiment lasted 1200 milliseconds, producing high-time-resolution pressure data capable of capturing even subtle fluid motion changes and transient characteristics.

Additionally, the system incorporated a bubble contamination simulation, since bubble formation within the pipeline can significantly disturb pressure signals and compromise the accuracy of anomaly detection. To identify and differentiate the influence of bubbles, BV1 and BP1–BP7 were designated as bubble injection and localization points, facilitating subsequent analysis of how bubble-induced disturbances differ from other types of faults.

In terms of operation, the experiment simulated the periodic opening and closing of solenoid valves to reproduce real switching behavior in propulsion systems. Specifically, each solenoid valve opened at 100 ms and closed at 300 ms, with a total cycle of 400 ms, repeated three times to complete the 1200 ms experimental duration. Although slight timing deviations of approximately ±1 ms might occur in actual control mechanisms, the overall cycles remained highly synchronized and consistent, ensuring comparability and accuracy in subsequent analyses.

Overall, despite being a simplified model, the experimental system was designed with both high fidelity and flexibility. It accurately represents transient pressure behaviors in propulsion processes while encompassing abnormal conditions such as bubble contamination, thereby providing a stable and diverse data foundation for fault detection and model validation in this study.

The training dataset covers three different spacecraft (Spacecraft #1 to #3) and includes three known conditions:

- Normal condition;
- Bubble anomaly;
- Solenoid valve fault.

In the training dataset, there are three spacecraft, each containing 35 normal cases, 16 solenoid valve fault cases, and 8 bubble anomaly cases, totaling 177 cases available for training. Specifically, Cases 1–105 correspond to normal conditions, Cases 106–153 to solenoid valve faults, and Cases 154–177 to bubble anomalies.

The training dataset comprises 177 sets of synthetically generated simulation data, with each set consisting of measurements from seven pressure sensors (labeled P1–P7).

**Figure 2:**
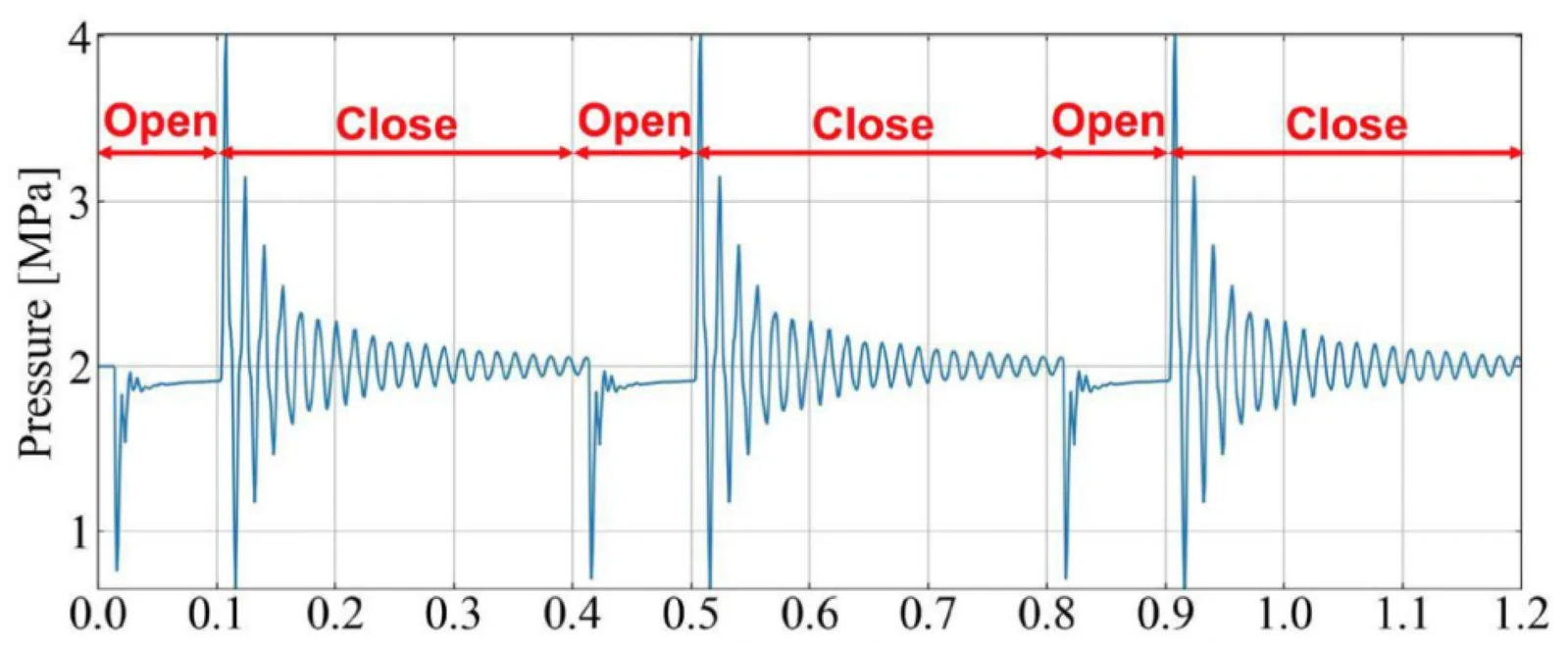
Pressure–time series of a typical case [3].

Figure 2 illustrates a typical pressure–time profile from one of the cases, depicting the system’s complete behavior under both steady-state and dynamic operating conditions. The propulsion system operates at a baseline pressure of approximately 2 MPa, with each test lasting 1.2 s. The sensors record pressure data at a sampling rate of 1 kHz, resulting in roughly 1200 instantaneous pressure samples per experiment.

The control strategy of the system follows a fixed-cycle design, with each cycle lasting 0.4 s, comprising two primary stages: valve opening and valve closing. In each cycle, the solenoid valve is activated (opened) at around 100 milliseconds and closed at 300 milliseconds, remaining open for approximately 200 milliseconds. During the subsequent interval, the system pressure naturally recovers to its steady state before entering the next operational cycle.

In the figure, three adjacent and uniformly periodic cycles can be clearly identified: 0–0.4 s, 0.4–0.8 s, and 0.8–1.2 s. Each time the valve transitions from closed to open or from open to closed, the abrupt change in fluid velocity and boundary conditions generates transient pressure fluctuations—commonly known as the water hammer effect. This phenomenon appears on the curve as pronounced peaks or troughs, followed by damped oscillations resulting from fluid–structure interactions within the pipeline. The oscillation amplitude gradually decreases until the system returns to a stable region near the initial pressure level. These dynamic pressure responses directly reflect the valve’s actual actuation characteristics, making them crucial for anomaly detection and health assessment.

In practical systems, component tolerances, actuation-signal jitter, and mechanical delays cause slight variations in valve timing, typically within ±1 millisecond. For instance, one opening event may occur at 99.7 ms instead of exactly 100 ms, or a closing event may be slightly delayed to 300.3 ms. Nonetheless, the control logic is designed to maintain overall cycle synchronization, keeping each total cycle stable at approximately 400 milliseconds. This small jitter yet highly synchronized control behavior ensures that data from different experiments remain aligned on a unified time axis, facilitating subsequent feature extraction and comparative analysis of key indicators such as pressure peaks, oscillation frequency, damping ratio, and recovery time constant. These parameters also serve as essential input features for machine learning models.

The test dataset contains 46 cases in total: Cases 178–200 correspond to Spacecraft #1, while Cases 201–223 belong to Spacecraft #4. It is important to note that Spacecraft #4 does not appear in the training dataset; thus, it is considered an unseen spacecraft during analysis, introducing higher levels of uncertainty and challenge. Moreover, the test set includes several previously unseen fault modes that do not exist in the training data. Therefore, the model must not only distinguish between normal, bubble, and solenoid valve faults, but also correctly classify unknown conditions. This design simulates real spacecraft missions, where new or unexpected anomalies may arise, serving as a rigorous test of the model’s generalization capability and practical applicability.

## 2. Materials and Methods

### 2.1. Overview of the Main Process

**Figure 3:**
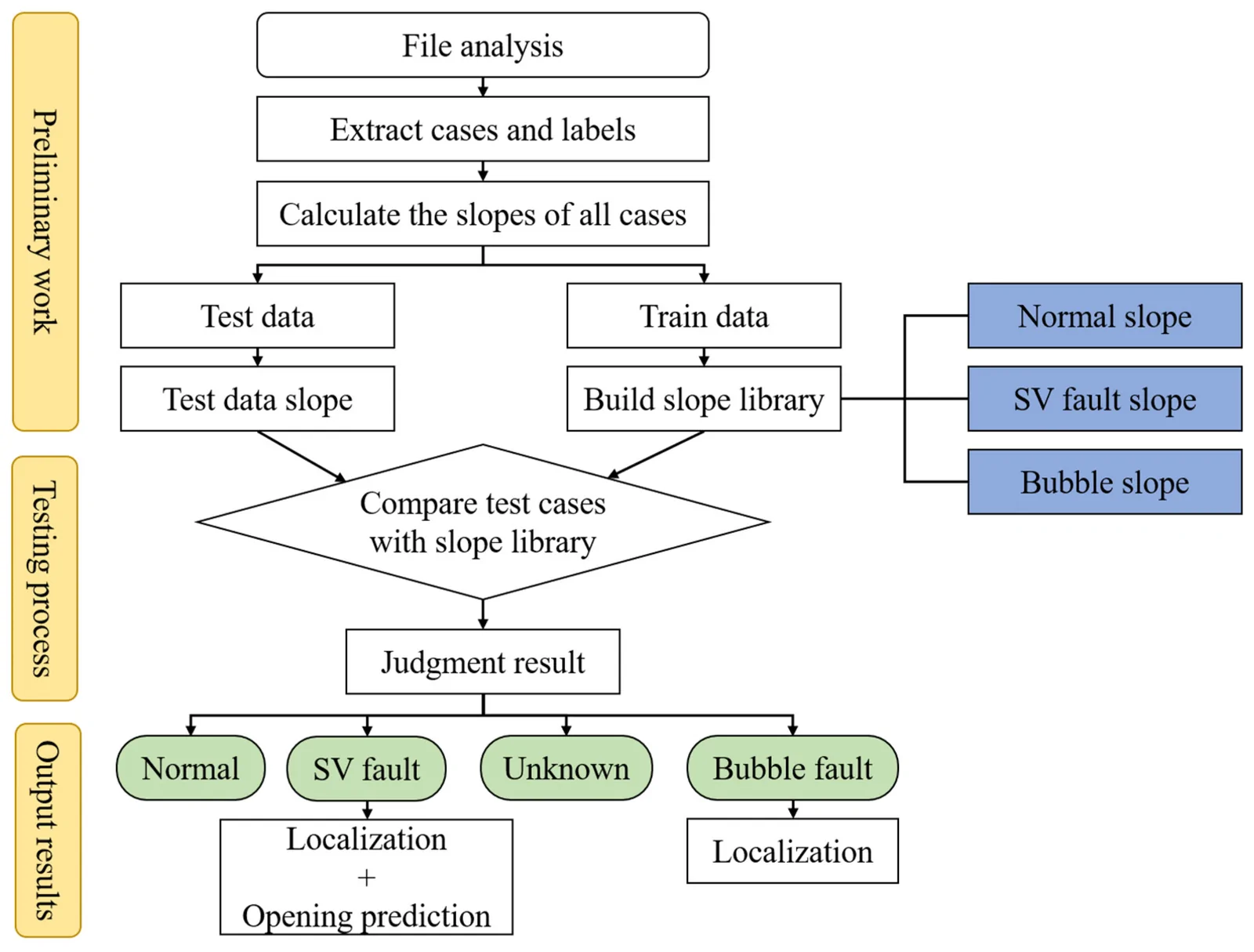
Flowchart of the proposed main process.

The method proposed in this study employs slope variation as the primary feature for data classification and valve-opening prediction. The overall framework is illustrated in Figure 3. The core concept lies in replacing high-dimensional statistical features with physically interpretable, simplified features, using slope analysis to capture the intrinsic dynamics of pressure changes within the system. This enables high-accuracy, low-complexity health-state identification. The approach offers both real-time capability and generality, making it particularly suitable for aerospace propulsion systems that require rapid feedback in industrial environments.

During the data preprocessing stage, the model automatically reads all case files within the dataset and loads their corresponding labels. Each case contains pressure data from seven sensors (P1–P7), and the model calculates the pressure–time slope for each sensor as the key feature describing system behavior. Unlike conventional methods that rely solely on instantaneous pressure values or time-series shapes, this study focuses on capturing the rate of pressure decrease to reveal the fluid’s true response at the exact moment of valve actuation, allowing more sensitive detection of when and where anomalies occur.

After slope extraction, the system constructs a standard slope library from the training data under various operating conditions (normal, bubble anomaly, and solenoid valve opening variations). In the testing phase, the model compares the slope behavior of unknown samples with the standard library. By calculating slope deviations and statistical distances, it determines whether the current system state deviates from the normal range. This classification process effectively distinguishes different operating modes of the spacecraft propulsion system, including Normal operation, Bubble Faults, and Solenoid Valve Faults (SV Faults).

When a bubble fault is detected, the system performs location identification by analyzing the relative slope changes of the seven pressure sensors, pinpointing the sensor that exhibits the earliest pressure drop. This enables inference of the bubble’s formation or passage point (BP1–BP7). If the model identifies a solenoid valve fault, it simultaneously carries out fault localization and opening prediction. Fault localization is based on the relative amplitude and rate of pressure drops across channels to determine the source of the anomaly, while opening estimation is achieved through a recursive slope-matching process. In this process, the slope of the test case is iteratively compared with the slopes from the training samples corresponding to different opening ratios (0%, 25%, 50%, 75%, and 100%), continuing until the error falls below a predefined threshold. This enables high-precision valve-opening estimation while maintaining computational stability.

Overall, this method establishes a unified slope-based quantification and comparison framework that achieves both accuracy and efficiency in health-state recognition. Compared with traditional time-alignment techniques such as DTW [4,16] or deep-learning-based approaches, it retains high classification performance while significantly reducing computational complexity and data requirements, thereby mitigating overfitting. More importantly, since the slope is a physically meaningful feature, it directly corresponds to engineering parameters such as pressure drop, valve flow area, and dynamic response rate, endowing the model with inherent physical interpretability.

In summary, this slope-oriented diagnostic framework not only distinguishes bubble and solenoid valve anomalies with high precision but also maintains robust prediction performance under previously unseen conditions, demonstrating excellent generalization ability. The proposed method holds strong potential for application in other fluid-control or aerospace subsystems as a core algorithm for predictive maintenance and health management, contributing significantly to improving spacecraft reliability and operational safety.

### 2.2. Parameter Selection and Slope Formulation

As shown in Table 1, although the solenoid valve activation occurs around 0.1 s, the DTW distances among all sample categories remain zero within the 0–0.1 s interval. This indicates that during this period, the pressure waveforms of all case types are nearly identical, exhibiting no significant variation or phase shift. Consequently, this time window demonstrates highly stable signal characteristics that effectively represent the system’s common initial state, unaffected by individual differences or noise. Therefore, the 0–0.1 s interval is particularly suitable as a reference segment for the classification model, serving as the standard baseline for distinguishing between normal and abnormal conditions.

Based on the above observations, this study selects the 0–0.1 s interval as the primary sampling window for feature extraction and slope calculation, aiming to capture the most physically meaningful stage of pressure variation during the initial valve-opening phase. Compared with full-time-domain analysis, this segment effectively focuses on the moment when fluid flow begins and the valve’s flow area changes rapidly, allowing the model to sensitively detect subtle anomalies inside the thruster. Meanwhile, this interval accounts for only 1/12 of the total recording duration, significantly reducing data volume and computational load without sacrificing informational completeness, thereby enhancing the real-time capability of classification and prediction.

To quantitatively describe pressure variation trends and establish a computable physical indicator, this study defines the pressure slope using a unified formula that characterizes the rate and direction of pressure change over a short period. This slope reflects both the fluid dynamic response and the valve actuation efficiency, serving as the core physical feature of the proposed method.

Specifically, the algorithm searches for the first occurrence of a pressure drop within the selected interval and takes the preceding data point’s pressure as the reference. By comparing the pressure difference and time difference between these two points, the local transient slope is obtained. If the pressure decrease is too small to indicate a meaningful flow change, the point is automatically excluded to prevent misclassification. This definition effectively captures the instantaneous response rate of the fluid under external stimuli—such as valve opening or bubble formation—and serves as the primary criterion for anomaly classification and valve-opening prediction.

(1) $$S=\frac{P\left(t_{i-1}\right)-P(t_{i})}{t_{i}-t_{i-1}}$$

Here, *P*(*t*) represents the pressure value at time *t*, *t*_0_ denotes the initial time of the data record, and *t_i_* is the first detected pressure drop point within the selected time window. This formula quantifies the rate of pressure change through the ratio of pressure difference to time difference, serving as a key input feature for subsequent classification and prediction models.

Since the slope feature directly reflects the magnitude, direction, and rate of pressure variation in the fluid’s dynamic process, it offers higher temporal resolution and dynamic sensitivity compared with conventional statistical features such as mean, peak value, standard deviation, or frequency-domain energy distribution. Empirical observations show that anomalies in propulsion systems typically occur at the exact moments when valves open or close. Analyses based solely on average or peak values fail to capture such brief yet critical changes in pressure. In contrast, the slope—representing the first derivative of pressure with respect to time—captures these rapid transitions on a millisecond scale, making it particularly effective for early anomaly detection.

For instance, in representative abnormal conditions:

- Bubble faults are often accompanied by a sudden pressure drop caused by gas formation or passage through the liquid, which disrupts fluid continuity and results in a local pressure collapse. This appears as a distinct negative slope peak on the slope curve.
- Solenoid valve opening faults, on the other hand, primarily affect the pressure recovery rate and steady-state behavior. Insufficient valve opening slows the pressure drop and prolongs the recovery time, producing smaller and smoother slope variations.

By comparing the slope trends from different sensors within the same time window, the model can identify both the type and location of anomalies. Furthermore, by matching these patterns with the corresponding normal and fault samples stored in the training database, the system achieves automated health-state classification and prediction.

The choice of the 0–0.1 s interval for slope analysis is also physically significant. This segment represents the critical transition from steady to transient flow, where pressure variations are most representative of valve-opening dynamics. Analyzing this specific period allows the model to focus on the most informative physical behavior while excluding the effects of later oscillations or external disturbances. The shortened analysis window also reduces computational cost, enabling faster, real-time diagnosis.

In summary, slope-based features offer clear advantages: computational simplicity, strong physical interpretability, and high anomaly sensitivity. Without relying on high-dimensional or frequency-domain transformations, they directly extract discriminative information from the raw time-domain signal, allowing the model to maintain high accuracy, stability, and real-time performance. This makes the approach particularly well-suited for aerospace propulsion system health monitoring and predictive maintenance, where rapid feedback is crucial. Moreover, the method can be extended to other fluid-control or hydraulic systems as a lightweight, physics-based state assessment strategy, demonstrating broad engineering applicability and future potential.

### 2.3. Detailed Testing Procedure

**Figure 4:**
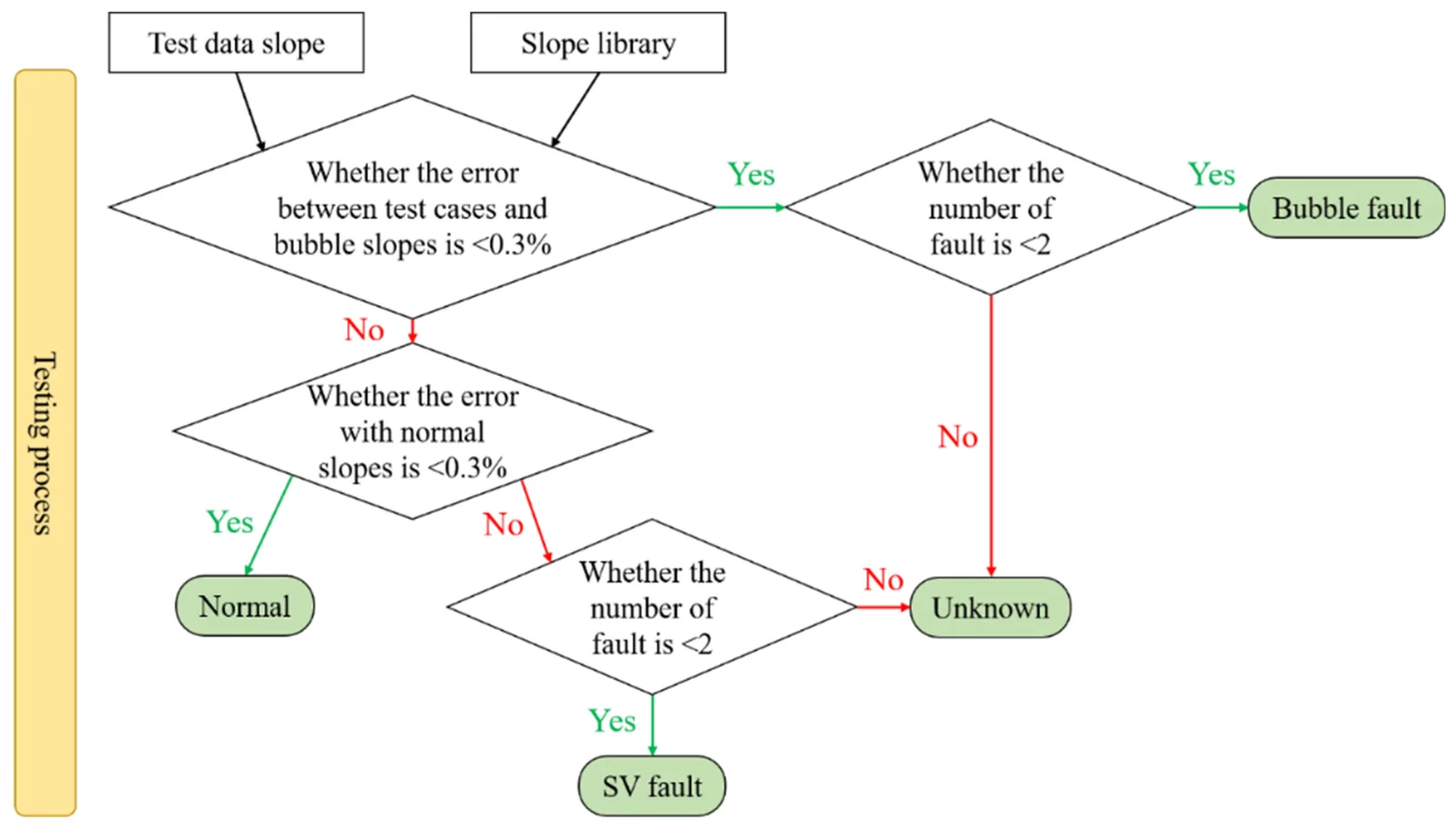
Flowchart of the testing procedure.

The testing procedure of this study, illustrated in Figure 4, is divided into three main stages: bubble fault detection, normal valve and solenoid valve fault determination, and unknown fault identification. The overall workflow aims to classify the operational state of the propulsion system based on slope features and to perform accurate, quantifiable fault diagnosis across different anomaly types. It is worth noting that, in order to reliably differentiate normal operating states from micro-bubble and solenoid-valve anomalies, a deviation threshold of 0.3% was established based on empirical signal characteristics analyzed strictly within the training dataset. This threshold explicitly accounts for inherent sensor noise uncertainty and minor ambient system fluctuations, serving as a robust noise floor.

I. Bubble Fault Detection

First, the pressure slopes of each test case are computed and compared with those stored in the bubble fault slope library.

- If the slope deviation between the test case and the bubble reference is less than 0.3%, the case is classified as a bubble fault, and the number of affected valves is recorded.
- If the deviation exceeds this threshold, the case proceeds to the next step for comparison with the normal slope library to determine whether it corresponds to another fault or a normal state.

II. Normal Valve and Solenoid Valve Fault Determination

For cases not identified as bubble faults, the computed slope is compared with the normal valve slope library.

- If the deviation between the test slope and the normal reference is less than 0.3%, the case is categorized as normal, indicating stable system operation.
- If the deviation exceeds 0.3%, the case is diagnosed as a solenoid valve fault, and the system identifies the number of faulty valves.

This step ensures that the model can effectively distinguish normal operation from solenoid valve malfunctions and provides detailed fault localization information.

III. Unknown Fault Identification

After completing the bubble and solenoid valve fault evaluations:

- If the detected number of bubble-fault or solenoid-fault valves is fewer than two, the case is categorized as a single-type fault (either bubble or solenoid).
- However, if two or more faulty valves are detected simultaneously, this indicates a multi-fault or compound anomaly that cannot be attributed to a single fault type. In such cases, the system classifies the sample as an unknown fault, prompting operators or maintenance personnel to conduct further investigation.

Through this three-stage process, the proposed method systematically identifies normal, bubble, solenoid, and unknown fault conditions, ensuring reliable diagnostic coverage and interpretability for spacecraft propulsion system health assessment.

Overall, the proposed testing procedure integrates slope features with tolerance-based comparison, forming a systematic and reproducible fault diagnosis mechanism. As illustrated in Figure 4, the workflow clearly demonstrates the complete operational sequence—from data input and slope computation to multi-type fault determination—ensuring both reproducibility and practical applicability of the testing method.

Figure 4 presents the testing flowchart, depicting the logical relationships and sequential steps involved in the entire diagnostic process, including data input, slope comparison, faulty valve counting, and the final classification decision. This provides an intuitive overview of the analytical framework proposed in this study.

In this study, all test cases are categorized into four types based on their slope features: Normal, Solenoid Valve Fault, Bubble Fault, and Unknown Fault. The classification criteria for each type are described below, and the overall classification process is illustrated in Figure 4.

1. Normal Range (Normal Condition)

A case is classified as Normal if its slope falls within the mean ± 0.3% range computed from the training data. This tolerance accounts for sensor measurement uncertainty and minor system fluctuations, ensuring that normal cases are accurately identified without being affected by small variations.

2. Solenoid Valve Fault (Fault Detection)

If a case’s slope does not belong to either the normal or bubble ranges, it is classified as a Solenoid Valve Fault, corresponding to valve identifiers SV1–SV4. The classification is based on the degree of slope deviation, allowing precise localization of the faulty valve and serving as the basis for subsequent valve-opening estimation.

3. Bubble Anomaly (Bubble Fault)

If a case’s slope matches the standard slope distribution established from bubble cases (Case 154–177) within an error range of ±0.3%, it is identified as a Bubble Fault. The corresponding sensor position (BP1–BP7) is then reported. This method enables accurate detection of bubble-related anomalies and provides valuable information for system maintenance and operational control.

4. Unknown Fault

If more than one faulty valve is detected in either the solenoid or bubble fault evaluation, it indicates the possible coexistence of multiple anomalies, making it impossible to classify the case under a single fault type. Such cases are therefore labeled as Unknown Faults, alerting operators to conduct further investigation and detailed diagnostics.

### 2.4. Opening Ratio Estimation

**Figure 5:**
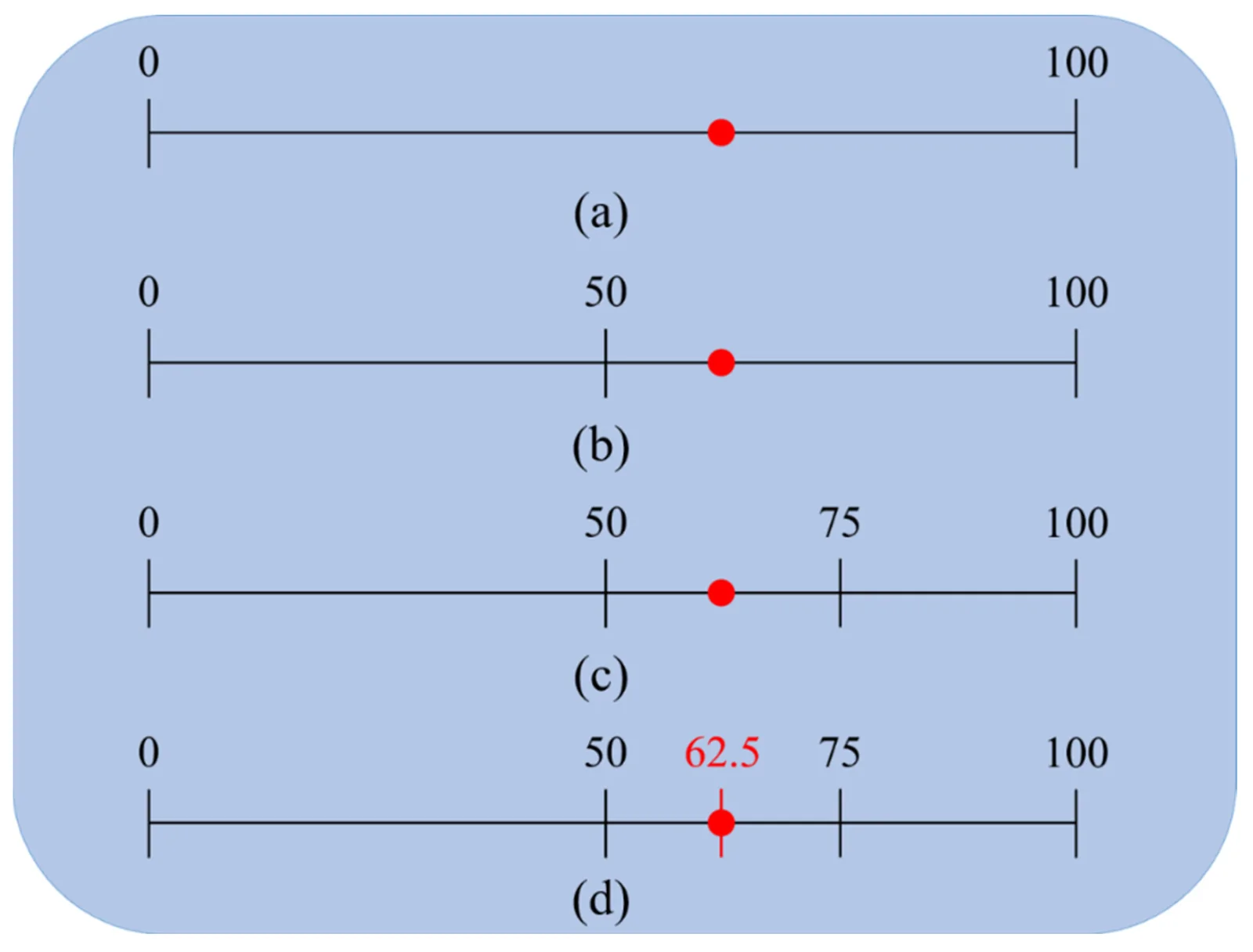
Schematic diagram of the recursive valve-opening prediction process.

For valves identified as faulty, their corresponding slopes are recursively compared with the slope–opening reference table constructed from the training data at 0%, 25%, 50%, 75%, and 100% opening levels. The comparison continues iteratively until the deviation falls below 0.01%, allowing precise estimation of the valve’s actual opening ratio. This process is illustrated in Figure 5.

As shown in Figure 5:

(a) The slope of the test case lies between the reference slopes corresponding to 0% and 100% openings.
(b) The interval 0–100% is first divided in half; the case slope is found to be greater than the 50% reference slope.
(c) The interval 50–100% is then halved again, and the case slope is determined to be smaller than the 75% reference slope.
(d) Finally, the interval 50–75% is subdivided, and when the difference between the case slope and the reference slope becomes less than 0.01%, the valve opening is precisely estimated as 62.5%.

Furthermore, the fine-grained 0.01% threshold implemented for valve-opening estimation was strictly parameterized to achieve high precision in resolving actual valve opening ratios. Maintaining this low tolerance limit prevents premature or erroneous opening detection caused by low-amplitude hydraulic variations. Raising this threshold expands the error margin, thereby compromising the estimation accuracy required to reliably characterize subtle operational changes in valve positioning.

## 3. Results

### Test Results

**Figure 6:**
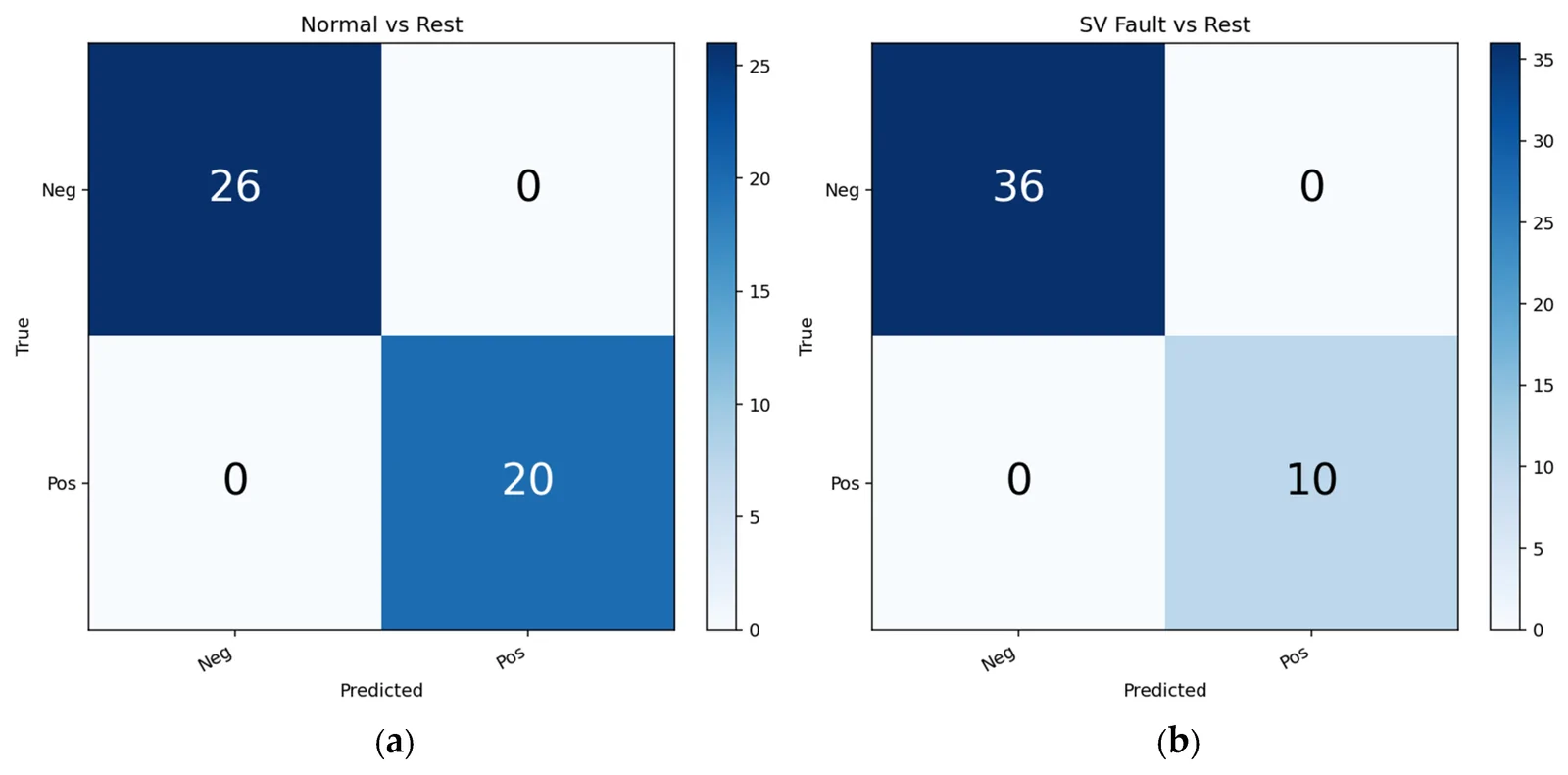

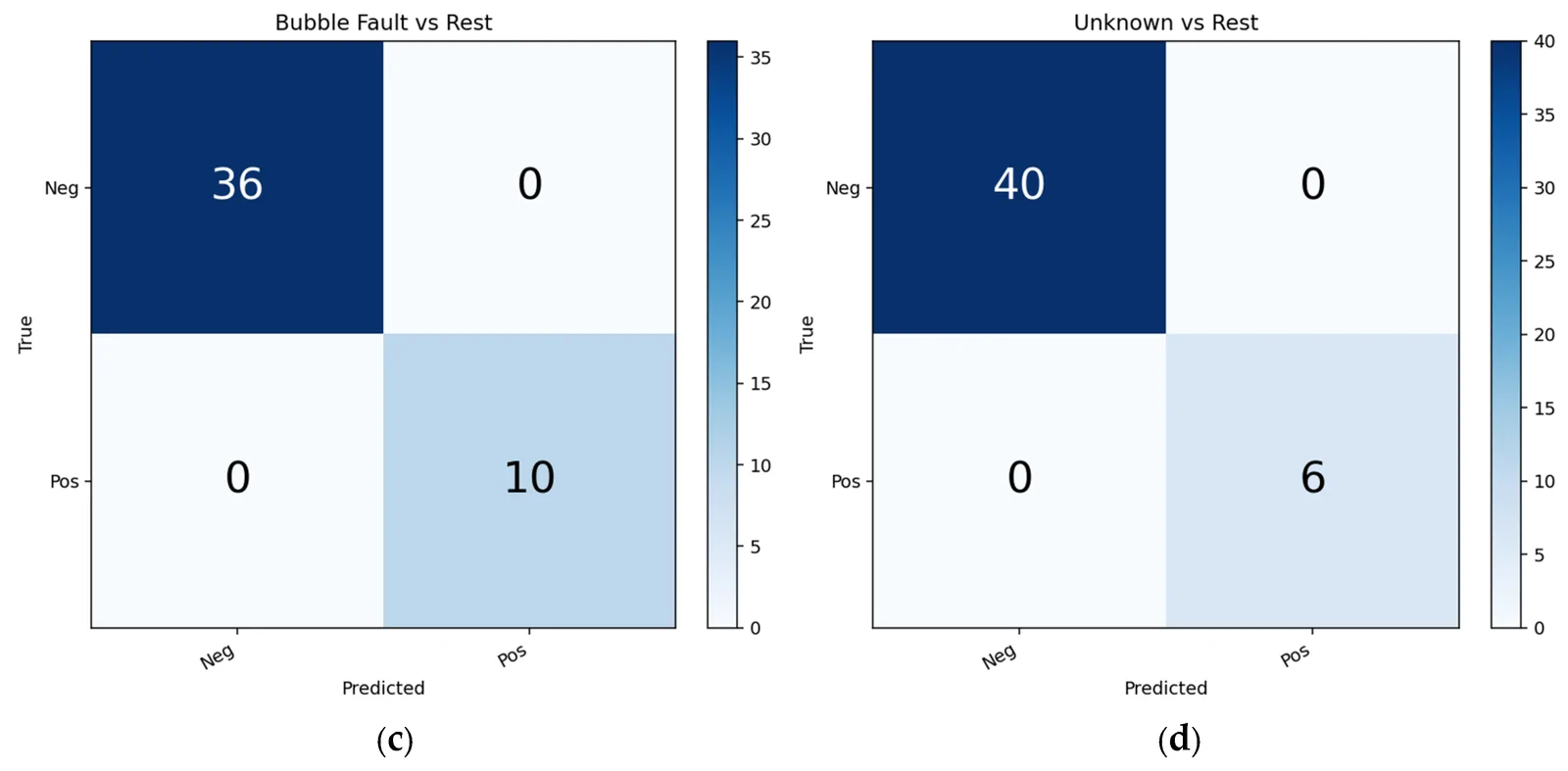
One-versus-rest confusion matrices for the 46 test cases: (**a**) Normal; (**b**) SV fault; (**c**) Bubble fault; (**d**) Unknown. The rows represent the true labels, and the columns represent the predicted labels. Pos denotes the target state, whereas Neg denotes all remaining states.

The proposed method successfully identified and classified all fault and anomaly locations with low computational cost while achieving an average absolute error (MAE) of only 0.59% [2] in valve-opening estimation, demonstrating a high degree of consistency with the ground truth. The classification results are shown in Figure 6.

The classification results presented in Figure 6 are evaluated using four one-versus-rest confusion matrices based on the same 46 test cases. For each confusion matrix, the target state is considered the positive class (Pos), while the remaining states are considered the negative class (Neg). The rows represent the actual labels, and the columns represent the predicted labels.

True positives (TPs) and true negatives (TNs) represent correctly classified cases, whereas false positives (FPs) and false negatives (FNs) represent incorrectly classified cases. To quantitatively evaluate the classification performance, four commonly used metrics are adopted: accuracy, precision, recall, and F1-score. These metrics are defined in Equations (2)–(5), respectively.

(2) $$\mathrm{Accuracy}=\frac{\left(TP+TN\right)}{\left(TP+TN+FP+FN\right)}$$

(3) $$\mathrm{Precision}=\frac{TP}{\left(TP+FP\right)}$$

(4) $$\mathrm{Recall}=\frac{TP}{\left(TP+FN\right)}$$

(5) $$F1\;\mathrm{score}=2\times \frac{Precision\times Recall}{\left(Precision+Recall\right)}$$

Using Equations (2)–(5), the classification performance metrics are calculated from the confusion-matrix values presented in Figure 6. The corresponding true positive (TP), true negative (TN), false positive (FP), and false negative (FN) counts, together with the calculated accuracy, precision, recall, and F1-score values, are summarized in Table 2.

Table 2 summarizes the confusion-matrix counts and classification metrics calculated using Equations (2)–(5). The true-positive and true-negative counts are 20 and 26 for Normal, 10 and 36 for SV fault, 10 and 36 for Bubble fault, and 6 and 40 for Unknown, respectively. All four one-versus-rest matrices have zero false positives and zero false negatives. Therefore, accuracy, precision, recall, and F1-score are 100% for each state across the same 46 test cases.

The proposed method still has room for improvement in valve-opening prediction. In particular, for Spacecraft #4, which lacks corresponding training data, the maximum prediction error reached 0.92%, while the minimum error was 0.02%. The detailed valve-opening prediction errors are summarized in Table 3.

Although slight deviations were observed in the valve-opening prediction, applying an unconditional rounding method to the decimal values within the model increases the prediction accuracy to 100%.

## 4. Discussion

This study proposed a fault classification and valve-opening prediction method centered on pressure slope features, applied to test cases of an aerospace propulsion system. The results show that the proposed method achieved 100% accuracy in operational state classification, effectively distinguishing between normal, bubble fault, solenoid valve fault, and unknown fault conditions, while accurately localizing the fault positions. In valve-opening regression prediction, the overall mean absolute error (MAE) was only 0.59%, and according to the official competition scoring scheme, the overall accuracy reached 99.74%, demonstrating high precision for cases within the range of training data coverage.

However, the study also revealed certain limitations of the model, particularly when handling data-scarce conditions such as those of the fourth spacecraft, where the maximum prediction error reached 0.92% [16]. This reflects the model’s insufficient generalization ability when facing operating conditions not represented in the training dataset. The finding suggests that, in practical applications, model performance may degrade when the operating environment significantly differs from the training domain.

Further, to guarantee the statistical integrity and generalizability of the proposed diagnostic framework, model development was executed under strict separation from the testing dataset. The slope reference library and classification thresholds were established using exclusively training set samples, ensuring that no information—either explicit features or implicit distributional properties—from the testing set was accessed, referenced, or indirectly incorporated during any phase of feature abstraction or threshold determination. Specifically, the construction of the slope reference library relied solely on the baseline state and known fault signatures derived from the designated training cases. All decision boundaries and classification parameters were finalized solely on the training data prior to testing, leaving the proposed method fully independent of testing labels. Furthermore, to eliminate potential sampling bias and ensure reproducibility, all reported performance metrics are derived from multiple repeated independent experiments. The consistent results across these repeated trials demonstrate that the proposed approach is robust, structurally sound, and entirely un-biased by the test data.

To reliably differentiate normal operating states from micro-bubble and solenoid-valve anomalies, a deviation threshold of 0.3% was established based on empirical signal characteristics analyzed strictly within the training dataset. This threshold explicitly accounts for inherent sensor noise uncertainty and minor ambient system fluctuations, serving as a robust noise floor. The generalization capability of this 0.3% threshold is validated by its consistent diagnostic stability on the completely unseen testing dataset. While the current implementation demonstrates high fidelity under this setting, a comprehensive quantitative sensitivity study—evaluating performance under adjacent boundary thresholds (e.g., 0.1%, 0.5%, and 1.0%)—will be incorporated in future work to fully map the operational trade-offs.

In summary, this research verifies the feasibility of using a single pressure-slope feature for both fault classification and valve-opening prediction. The method is simple and computationally efficient and possesses strong potential for real-time monitoring and predictive maintenance applications. Furthermore, the study identifies promising future directions, including data augmentation, multi-condition training, and model architecture optimization, to enhance the model’s generalization performance across different spacecraft and operating environments.

## 5. Conclusions

This study proposed an anomaly detection method based on a unified slope formula, capable of effectively distinguishing among normal conditions, bubble anomalies, and solenoid valve faults while accurately estimating the valve-opening ratio of faulty valves. Using the PHMAP 2023 test dataset, the proposed method achieved a 100% classification accuracy, with a maximum opening estimation error of only 0.92%, demonstrating its superior performance and reliability. Compared with other approaches, the proposed method offers advantages of low computational complexity, strong interpretability, and high potential for real-time implementation. Future work will focus on validating the approach under conditions involving high-frequency noise, multiple simultaneous anomalies, and real spacecraft data, as well as integrating machine learning models to enhance generalization capability in complex operational scenarios. The research significance and value of this study demonstrate the following values and contributions: (1) Academic field: This research proposes a low-cost and precise valve assessment method centered on pressure slope features, capable of effectively performing both fault classification and valve-opening prediction. The method introduces a novel perspective on feature selection and analysis within the fault diagnosis domain. It provides valuable insights for the development of diagnostic techniques in propulsion systems and similar fluid-control systems, serving as both a theoretical foundation and practical reference for future studies in this field. (2) Application field: The proposed method can be applied to smart manufacturing and industrial automation systems, offering real-time monitoring and predictive maintenance capabilities. By accurately identifying valve faults and anomaly locations, the system can significantly reduce operational risks and maintenance costs caused by valve malfunctions, thereby enhancing equipment reliability and production efficiency.

For future research works, it is worth noting that, although the proposed method provides an efficient framework with low computational burden, direct quantitative comparisons against alternative paradigms—such as Dynamic Time Warping (DTW), classical machine learning classifiers, and deep-learning architectures—were limited by the novelty of the dataset and sparse literature benchmarks. To comprehensively address these comparative trade-offs, future work will focus on implementing rigorous multi-metric benchmarking against these baselines, systematically evaluating not only classification accuracy but also memory footprint, training/inference complexity, and execution latency. Moreover, the current method still faces challenges in scenarios involving multiple simultaneous valve or bubble faults. Future research should include generalization to an unseen simulated spacecraft; generalization to unseen simulated fault conditions; and generalization to real spacecraft hardware. The latter should be presented as a future validation requirement rather than as an established capability.

## Figures and Tables

**Table 1:** In-class DTW distance (mean ± standard deviation) [4].

Conditions	0.0–0.1 s	0.1–0.4 s	0.4–0.5 s	0.5–0.8 s	0.8–0.9 s	0.9–1.2 s
Normal	0.0 ± 0.0	129.7 ± 59.3	11.3 ± 4.6	125.2 ± 58.2	11.6 ± 4.3	130.5 ± 61.0
Bubble: BP1	0.0 ± 0.0	157.2 ± 31.0	13.7 ± 4.9	156.1 ± 32.8	14.8 ± 2.7	152.1 ± 34.7
Bubble: BP2	0.0 ± 0.0	120.5 ± 25.5	6.1 ± 2.2	116.9 ± 22.9	5.2 ± 1.7	117.2 ± 23.1
Bubble: BP3	0.0 ± 0.0	118.3 ± 22.0	5.8 ± 1.6	115.8 ± 20.3	4.1 ± 0.9	115.1 ± 20.2
Bubble: BP4	0.0 ± 0.0	135.4 ± 58.5	7.8 ± 4.7	125.8 ± 51.7	10.7 ± 6.7	136.9 ± 59.6
Bubble: BP5	0.0 ± 0.0	144.2 ± 40.1	17.7 ± 9.2	143.7 ± 40.0	13.5 ± 6.4	145.9 ± 41.3
Bubble: BP6	0.0 ± 0.0	108.0 ± 21.8	3.6 ± 1.0	108.8 ± 21.6	3.3 ± 0.4	108.5 ± 21.6
Bubble: BP7	0.0 ± 0.0	135.4 ± 40.6	2.9 ± 0.6	147.3 ± 30.6	11.2 ± 5.8	152.0 ± 32.8
Bubble: BV1	0.0 ± 0.0	182.8 ± 27.8	14.3 ± 3.3	167.7 ± 26.6	15.2 ± 3.3	159.8 ± 25.7

**Table 2:** Confusion-matrix counts and classification performance metrics derived from Figure 6.

STATE	TP	TN	FP	FN	ACC. (%)	PREC. (%)	REC. (%)	F1 (%)
NORMAL	20	26	0	0	100	100	100	100
SV FAULT	10	36	0	0	100	100	100	100
BUBBLE FAULT	10	36	0	0	100	100	100	100
UNKNOWN	6	40	0	0	100	100	100	100

**Table 3:** Valve-opening prediction error table.

CASE	GROUND TRUTH	PREDICTION	ERROR (%)
179	22%	22.46%	0.46
181	76%	76.37%	0.37
188	5%	5.66%	0.66
190	46%	46.68%	0.68
199	98%	98.24%	0.24
202	44%	44.73%	0.73
205	94%	94.92%	0.92
211	95%	95.90%	0.90
212	70%	70.90%	0.90
214	24%	24.02%	0.02

## Data Availability

The data presented in this study are available in PHMAP 2023 Available online: https://phmap.jp/program-data/ (accessed on 1 January 2026) reference number [3]. These data were derived from the following resources available in the public domain: https://phmap.jp (accessed on 1 January 2026).

## Abbreviations

The following abbreviations are used in this manuscript:

PHMAP	Prognostics and Health Management Asia-Pacific
DTW	Dynamic Time Warping
MAE	Mean Absolute Error
SV	Solenoid Valve
